# Prefabricated resin veneer: A case report of a simplified restorative technique

**DOI:** 10.15171/joddd.2018.022

**Published:** 2018-06-20

**Authors:** Pedro Paulo Albuquerque, Marina Barreto Pereira Moreno, Alexander Cassandri Nishida, Ezequias Rodrigues, Camila Kiyohara, Carlos Eduardo Francci

**Affiliations:** ^1^Department of Biomaterials and Oral Biology, São Paulo Dental School, University of São Paulo – USP, São Paulo, SP, Brazil; ^2^Department of Dental Materials, Piracicaba Dental School, University of Campinas – UNICAMP, Campinas, SP, Brazil; ^3^Department of Dentistry, São Paulo Dental School, University of São Paulo – USP, São Paulo, SP, Brazil

**Keywords:** Dental veneer, dental resin, composite resin

## Abstract

***Background:*** the aim of the study was to describe the step-by-step of clinical cases using prefabricated composite resin veneers (PCRVs), manufactured with the composite Brilliant New Generation (Coltene, Altstätten, Switzerland). Direct composite veneers presented some drawback as the difficult of execution and color instability of the composite over the time. The simplified application of the PCRVs presented as an interesting alternative in cases of smile asymmetry, large deficient restorations and discolored tooth. In the present investigation, the complete description of the PCRVs technique can help the dentist during the planning and execution of treatments with the Componeer system.

***Conclusion:*** the treatment with Componeer Brilliant NG showed excellent aesthetic results. PCRVs technique is simpler than direct composite veneers. The specific characteristics of the system can promote results with greater aesthetic longevity. It is important to highlight that this procedure does not replace the already established veneer technique with dental ceramics.

***Clinical implication:*** PCRVs presented an excellent surface gloss. Their dimensions based on the golden ratio facilitate the technique and turns as an excellent alternative in the aesthetic restorative treatment in the anterior region.

## Introduction

Dental veneers have become an attractive treatment in dentistry spurred by the development of different materials and techniques, associated with the aesthetic patterns imposed by society.^1^ In general, patients that presented clinical scenarios in the anterior teeth such as increased interdental spaces, fractures, deficient restorations or color changes are indicated for the treatment with veneers.^2,3^

The success of the veneers is associated with the dentist’s knowledge, the technique used, the restorative material (dental ceramics and composite resin) and the patient’s collaboration.^4^ The ceramic veneers were proposed by Dr. Charles Pincus in 1938.^5^ Since then, the treatment with ceramic veneers has become one of the greatest themes in Dentistry due to the excellent aesthetic results. Ceramics are biocompatible and exhibit high wear resistance and great color stability. Nowadays, ceramic is the main restorative material used in veneer treatments.^8,9^ However, some specific properties such as brittleness and the superior hardness of the ceramic in relation to the dental tissue are considered the disadvantages for this class of restorative materials. Additionally, the high cost of the ceramic prevents some patients from proceeding with the treatment.^10,11^

Restorative protocols with direct composite veneers have been introduced as an alternative for patients who cannot afford the high inherent cost of ceramics. Although cheaper, this technique presents some drawbacks, including the difficulty in the mirroring, color matching, construction of structures (e.g. dentin mamelons and enamel characteristics such as translucency/opalescence) and the incorrect reproduction of the dental surface texture.^12^ Additionally, the time required to execute the resin veneer, the color instability of the material, marginal infiltration and secondary caries are also critical factors that might intimidate the dentist to carry out the technique.^8,12^

The PCRVs simplifies the veneer technique and their properties can improve the treatment longevity.^13^ The PCRV Componeer is manufactured from the composite Synergy D6 or Brilliant NG, both nanohybrid materials. This PCRVs are fabricated under controlled laboratory conditions in relation to light, pressure and temperature. After these treatments, the material exhibits improved polymerization, reaching a higher degree of conversion with lower pores and internal defects. PCRVs are available for the anterior teeth with 0.3‒1.0 mm of thickness with different sizes (small, medium, large and extra-large), two shades (transparent and bleach) and proportions based on the golden ratio concepts.^13,14,15^

In the reviewed literature, there are no clinical studies reporting the use of PCRV fabricated with the Brilliant NG composite resin. Only works with the PCRV produced with the Synergy D6 composite resin are available. According to the manufacturer, the Brilliant NG resin has a different organic matrix compared to Synergy D6, which might influence the final behavior of restorations over time. Therefore, the aim of the study was to describe two cases (step-by-step) with PCRV fabricated with the Brilliant NG resin.

### Case reports

### CASE 1 – Single tooth restored with a PCRV

**Figure 1:**
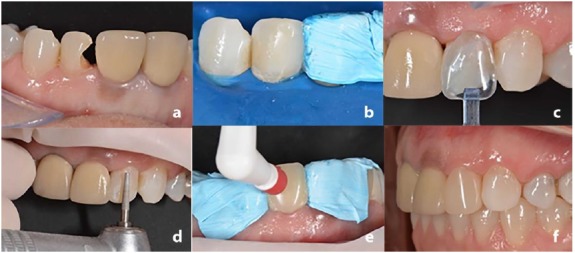
Case 1. Initial aspect (a). Direct restoration with Brilliant NG composite (A2/B2 dentin; b). Size selection with the Contour Guide (c). Tooth reduction to receive the PCRV (d). Placer Instrument of the Componeer system (e). Final aspect (f).

A 42-year-old male patient presented at the clinic with a debonded restoration in tooth #22 (Figure 1a). A clinical examination revealed acceptable periodontal condition and no carious lesions. After analyzing the size of the restoration and the desire of the patient in solving the problem, rehabilitation with PCRV (Componeer - Brilliant NG) was proposed.

The color matching was performed with a color shade guide of the PCRV system, and the A2/B2 dentin shade associated with the veneer (transparent) was selected. The color matching of the Componeer relies on the concept of natural layering, in which two layers of the incremental technique is able to mimic the natural aspect of the teeth. Moisture was controlled with a rubber dam and a new restoration was placed on #tooth 22 to reestablish the original anatomy (Figure 1b). The treatment proceeded with the selection of the PCRV size (medium), using the contour guide specific to the Componeer (Figure 1c). This contour guide presented different sizes of PCRV (small, medium, large and extra-large) for the antero-superior and inferior tooth. The dentist can always select the correct size for each patient, respecting the fundamentals of the aesthetic smile.

A minimal preparation was performed on the tooth buccal surface with a diamond bur #2068 (KG Sorensen, Cotia, Brazil) to facilitate the setting of the PCRV (Figure 1d). The dental wear did not involve dentin. It is important to highlight that there is no specific amount of dental wear for luting of a PCRV, and the dentist should evaluate minimal wear to facilitate the luting procedure. The dental substrate was etched with 37% phosphoric acid (Magic Acid, Coltene) for 30 seconds, followed by abundant water rinse and air drying. The adhesive system One Coat Bond (Coltene) was applied with a Technobrush (Coltene) on the tooth and on the internal surface of PCRV. The Brilliant NG composite resin (A2/B2 dentin) was used as the luting agent. Clinical steps of tooth wear, adhesive procedure and cementation were executed without the use of a rubber dam. However, it is important to note that the control of moisture was ensured through the insertion of the retraction cord (Pro Retract 0000 FGM, Joinville, Brazil). Such technique allows a satisfactory control of the gingival fluid and facilitates the correct positioning of PCRV.

The veneer was fixed on the tooth with the instrument “Placer” included in the Componeer system (Figure 1e). Excess resin was removed after a slight compression of the PCRV. The light-curing was carried out with an LED (Radii cal, SDI, Bayswater, Victoria, Australia) with a irradiance of 1.200 mW/cm^2^ for 40 seconds. The excess resin was removed and no final polishing was required due to manufacturers’ pre-polishing of the PCRV (Figure 1f). The patient was extremely pleased with the result.

### CASE 2 – Multiple restorations with PCRV

**Figure 2:**
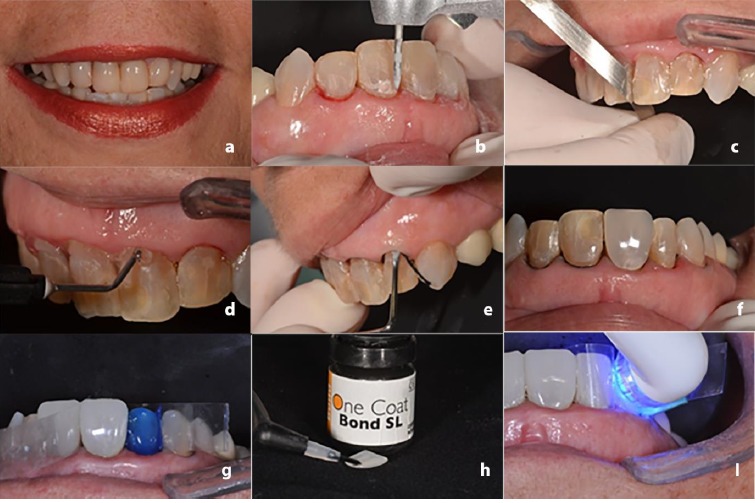
Case 2. Initial aspect (a). Preparation of tooth (b). Interproximal wear with abrasive stainless-steel strips (c). Application of hemostatic gel (d). Insertion of retraction cord (e). Dry proof of PCRV (f). Acid etching (g). Application of adhesive on the PCRV (h). light-curing with LED (i).

In addition to single element (case 1), the PCRVs are also indicated for extensive rehabilitations. A 37-year-old female patient presented at the clinic, unhappy with her smile. Clinical examination revealed some deficient restorations (class III and IV) with multiple color changes (Figure 2a). As the patient requested urgency in the treatment, a one-visit technique using PCRVs was suggested.

The restorations were removed with diamond burs (Figure 2b), followed by the relief of the interproximal contact with abrasive stainless-steel strip (Figure 2c). The teeth #13-23 were submitted to a minimum wear (0.5 mm) with a diamond bur in the buccal surface to facilitate the PCRV insertion. A hemostatic gel (Hemosthase, FGM, Joinville, Brazil) was applied in the gingival margin to contain possible bleeding (Figure 2d), and a single retractor cord (ProRetract 0000, FGM, Joinville, Brazil) was inserted to control moisture (Figure 2e).

Subsequently, the contour guide was used to select the veneer size (medium) and a dry proof with the PCRV was performed to view the possible final result (Figure 2f). The color matching was performed as described in case 1, and the Brilliant NG composite resin (A1/B1 dentin) and PCRV (Bleach) were selected. A mylar strip (Epitex, GC, Alsip, EUA) was inserted into the proximal regions and fixed with a wood wedge (TDV, Santa Catarina, Brazil) to facilitate cervical adaptation during cementation. The teeth were etched for 30 seconds with 37% phosphoric acid (Magic Acid, Coltene) (Figure 2g), followed by abundant rinsing and drying. The adhesive One Coat Bond (Coltene) was applied with a Technobrush (Coltene) and gently air-dried in order to remove the excesses. The same adhesive system was also applied on the internal surface of the PCRV (Figure 2h).

**Figure 3:**
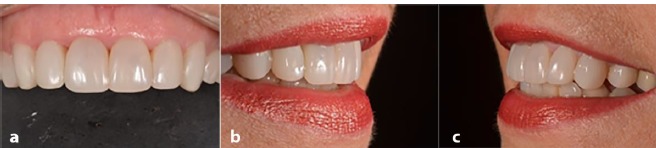
Final aspect of case 2. Facial view (a). Right side view (b). Left side view (c).

The Brilliant NG composite resin (A1/B1 dentin) was manipulated in a sterile glass plate to facilitate the manipulation and adaptation into the PCRV. An instrument of the Componeer system was used to seat the PCRV and the excess resin was removed. Photoactivation was performed as described in case 1 (Figure 2i). Cervical and proximal polishing was performed with abrasive disks (Diamond Pro, FGM), felts and diamond paste. The patient was very happy with the result. It was possible to observe the harmonic smile of the patient, with the alignment of the teeth and the absence of differences in color shade (Figure 3).

## Discussion

Different techniques are available for anterior restorations. The design of the tooth preparation can be extensive (e.g. total crown) or minimally invasive (e.g. veneers).^5^ Although different, both crown and veneer treatments require multiple clinical and laboratory steps. Therefore, the single session required in the treatment with PCRV has attracted a lot of attention in the dental community.^17,19,20,21^

The ceramic veneers have been considered the gold standard due to their well-known properties.^8,9^ A previous study reported high survival rate of ceramic veneers, especially when the luting procedure is limited to enamel tissue.^9^ Additionally, the literature reported that the ceramic type and the extension of dental wear corroborate to the satisfactory results obtained.^2,3,9^ A well-known veneer alternative is based on the use of direct composite resin. This technique provides a high reproduction of details due to the greater number of composite with different shades. However, some drawbacks such as color instability associated with the difficulty to execute the restoration, render this technique a hard work for the dentist. In the present study the use of PCRVs was simple and effective in meeting the patient’s need in only one session.

The use of indirect composite veneer is not new.^8^ This procedure was proposed with the Mastique Laminate Veneer (Caulk, Milford, DE, USA) in the 1970s.^23^ At this time, the veneers were produced with acrylic resin and the luting procedure was carried out using a light-sensitive composite resin. Although innovative, the inherent fragility of the acrylic veneers associated with the weak bonding to the dental tissue resulted in the failure of the system.

Recently, two systems of PCRV were proposed. The Direct Veneer (Edelweiss, Wolfurt, Austria) was developed in 2009 and the Componeer (Coltene) in 2011.^24^ Both systems presented PCRVs produced under laboratory conditions. It is inferred that the treatment used in the manufacturing of the Componeer could result in a high degree of conversion of the composite. In theory, this polymerization can promote an increase in the density of cross-linked double bonds into the polymer network. This reaction can decrease the amount of non-reactive components, resulting in a material with high strength, hardness and color stability in relation to direct photo activated composites.^14,22^

Previous studies reported some difficulties in the color matching of direct resin restorations.^13,22^ An interesting feature of the Componeer system (Brilliant NG) is presented by the chameleon effect of the composite used for the veneer cementation. It is reported that this technology induces a selective refraction index of the inorganic particles in the composite formulation, which provides a lower number of the color since the composite is able to reproduce different shades of the VITA scale (e.g. Brilliant NG A1/B1 dentin).^13,16^ This characteristic was confirmed in the present investigation and just one composite shade was used for the luting of the PCRV. No color difference can be observed in the final result.

The literature reported several indications for PCRV.^13-16^ Previous clinical studies described the use of PCRV manufactured with the Synergy D6 composite resin to solve different clinical scenarios. Aesthetic reestablishment of the smile,^16,17^ elimination of tooth darkness resulting from an endodontic treatment^16,18-20^ and the correction of fluorosis stains have been reported with satisfactory results.^21^ However, fewer in vitro studies regarding the properties of PCRV are published in the literature. A previous work reported different results of bond strength for different materials.^22^ The PCRV and the ceramic E.max Press (Ivoclar Vivadent, Schaan, Liechtenstein) showed similar micro-shear bond strength after thermocycle aging. Despite the results obtained, it is important to observe that the author used different luting agents, which could affect the comparation of the final bond strength results.

The longest follow-up reported with the PCRV was no more than one year.^17^ In relation to the composite resin, the literature reported a decrease in the surface gloss over time. Therefore, a new polish procedure is necessary to reestablish the original aesthetic appearance. Thus, despite of the excellent immediate results obtained in the present study, further clinical studies with longer follow-up should be developed associated with laboratory researches in order to evaluate the mechanical and optical properties of the PCRV.

## Conclusion

Prefabricated composite resin veneers have been advocated as an alternative to direct composite veneers. The simplified execution and improved properties of the PCRV enable results with greater longevity. It is important to highlight that this procedure does not replace the already established veneer technique with dental ceramics.

## Acknowledgment

The authors are grateful to Coltene (Brazil) for the donation of the materials used in the described cases.

## Authors’ contributions

All authors contributed to the case selection, and treatment planning. PPA rendered the treatments, and drafted the manuscript. All authors have contributed to the critical revision of the manuscript, and have read and approved the final manuscript.

## Funding

Not applicable.

## Competing interests

The authors declare no competing interests with regards to the authorship and/or publication of this article.

## Ethics approval

The individuals whose information is included here have given written informed consents for the publication of this paper.

## References

[1] Moraschini V, Fai CK, Monte Alto R, Santos GO (2015). Amalgam and resin composite longevity of posterior restorations: A systematic review and meta-analysis. J dent.

[2] Peumans M, Van Meerbeek B, Lambrechts P, Vanherle G (2000). Porcelain veneers: a review of the literature. J Dent.

[3] Albanesi R B, Pigozzo MN, Sesma N, Lagana DC, Morimoto S (2016). Incisal coverage or not in ceramic laminate veneers: A systematic review and meta-analysis. J Dent.

[4] Francci CE, Riquieri H, Nishida AC, Saavedra GSFA (2016). Harmonização do sorriso – Do planejamento digital à cimentação de laminados Prepless – Parte I. Eur J EsthetDent.

[5] Pincus CR. Building mouth personality. A paper presented at: California State Dental Association 1937; San Jose, California.

[6] Van Meerbeek B, Yoshihara K, Yoshida Y, Mine A, De Munck J, Van Landuyt KL (2011). State of the art of self-etch adhesives. Dent Mater.

[7] Pashley DH, Tay FR, Breschi L, Tjaderhane L, Carvalho RM, Carrilho M, Tezvergil-Mutluay A (2011). State of the art etch-and-rinse adhesives. DentMater.

[8] Demarco FF, Collares K, Coelho-de-Souza FH, Correa MB, Cenci MS, Moraes RR, Opdam NJ (2015). Anterior composite restorations: A systematic review on long-term survival and reasons for failure. Dent Mater.

[9] Gurel G, Sesma N, Calamita Ma, Coachman C, Morimoto S (2013). Influence of Enamel Preservation on Failures Rates of Porcelain Laminate Veneers. Int J Periodontics Restorative Dent.

[10] Anusavice KJ (2012). Standardizing failure, success, and survival decisions in clinical studies of ceramic and metal-ceramic fixed dental prostheses. Dent Mater.

[11] Rashid H, Sheikh Z, Misbahuddin S, Kazmi MR, Qureshi S, Uddin MZ (2016). Advancements in all-ceramics for dental restorations and their effect on the wear of opposing dentition. Eur J Dent.

[12] Heintze SD, Rousson V, Hickel R (2015). Clinical effectiveness of direct anterior restorations—A meta-analysis. Dent Mater.

[13] Rusher G. Direct restoration of lower anteriors with componeer by Coltene/Whaledent. User- Report 2011:COMPONEER.

[14] Gomes G, Perdigao J (2014). Prefabricated composite resin veneers--a clinical review. J of Est and Rest Dent: official publication of the American Academy of Esthetic Dentistry.

[15] Shinde TV, AS  D (2014). Componeers crowning glory of esthetic dentistry. Int J Dental Clin.

[16] Shumilovich BR, Spivakova IA, YB YB (2014). V Clinical Experience with a System of Direct Componeer (Coltene/Whaledent, Switzerland) Composite Veneers Work Difficulties and Ways of Overcoming Them. J Health Sci.

[17] Martini CE, Parreiras SO, Sezsz AL, Pupo YM, Gomes GM, Mongruel OMG, Gomes  JC (2016). Aesthetic Treatment with Prefabricated Composite Veneers– Case Report. Dent O Cran Res.

[18] Dietschi D, Devigus A (2011). Prefabricated Composite Veneers: Historical Perspectives, Indications and Clinical Application. Eur J EsthetDent.

[19] Gonçalves R, Correia I, Cardoso Ferreira J, Pires P, Carvalho MT, Pina‐Vaz I (2015). Descoloração dentinária: aplicação de facetas Componeer®. Rev Port Estomatol Cir Maxilofac.

[20] Migliau G, Besharat LK, Sofan AAA, Sofan EAA, U  R (2015). Endo-restorative treatment of a severely discolored upper incisor: resolution of the “aesthetic” problem through Componeer veneering System. Ann Stomatol.

[21] Du Toit J, Patel N, Montalli V, S J (2012). Aesthetic treatment of severely fluorosed teeth with prefabricated composite veneers: a case report. IntDent–AfricanEdition.

[22] Perdigao J, Sezinando A, Munoz MA, Luque-Martinez IV, Loguercio AD (2014). Prefabricated veneers - bond strengths and ultramorphological analyses. J Adhes Dent.

[23] Haas BR (1982 Fall). Mastique veneers: a cosmetic and financial alternative in post-periodontal care. J N J Dent Assoc.

[24] Dietschi D, Devigus A (2011). Prefabricated Composite Veneers Historical Perspectives Indications and Clinical Application. Eur J Esthet Dent.

